# Pooled prevalence and determinants of informed choice of contraceptive methods among reproductive age women in Sub-Saharan Africa: A multilevel analysis

**DOI:** 10.3389/fpubh.2022.962675

**Published:** 2022-09-14

**Authors:** Nuhamin Tesfa Tsega, Tsion Tadesse Haile, Melaku Hunie Asratie, Daniel Gashaneh Belay, Mastewal Endalew, Fantu Mamo Aragaw, Sintayehu Simie Tsega, Moges Gashaw

**Affiliations:** ^1^Department of Women's and Family Health, School of Midwifery, College of Medicine and Health Sciences, University of Gondar, Gondar, Ethiopia; ^2^Department of General Midwifery, School of Midwifery, College of Medicine and Health Sciences, University of Gondar, Gondar, Ethiopia; ^3^Department of Human Anatomy, College of Medicine and Health Sciences, University of Gondar, Gondar, Ethiopia; ^4^Department of Epidemiology and Biostatistics, Institute of Public Health, College of Medicine and Health Sciences, University of Gondar, Gondar, Ethiopia; ^5^Department of Environmental and Occupational Health and Safety, Institute of Public Health, College of Medicine and Health Sciences, University of Gondar, Gondar, Ethiopia; ^6^Department of Medical Nursing, School of Nursing, College of Medicine and Health Sciences, University of Gondar, Gondar, Ethiopia; ^7^Department of Physiotherapy, School of medicine, College of Medicine and Health Science, University of Gondar, Gondar, Ethiopia

**Keywords:** pooled, determinants, informed choice of contraceptive methods, multilevel analysis, Sub Saharan Africa

## Abstract

**Background:**

Despite the commitments of the government to minimize unintended pregnancy, abortion, and unmet need for contraceptives, as per our search of the literature, there is no study on the pooled prevalence and determinants of informed choice of contraceptive methods in sub-Saharan Africa. Therefore, this study aimed to assess the pooled prevalence and determinants of informed choice of contraceptive methods among reproductive-aged women in sub-Saharan Africa.

**Methods:**

This study was based on the 32 Sub-Saharan African countries Demographic and Health Survey data. A total weighted sample of 65,487 women aged 15–49 was included in the study. The data were cleaned, weighted, and analyzed using STATA Version 14 software. Multilevel logistic regression modeling was used to identify determinants of an informed choice of contraceptive methods. Adjusted odds ratio (AOR) with 95% Confidence Interval (CI) and *p*-value < 0.05 were used to declare the significant determinants.

**Result:**

The pooled prevalence of informed choice of contraceptive methods among reproductive age (15–49) women in sub-Saharan Africa was 49.47% (95%CI: 44.33, 54.62%) with *I*^2^ =99.5%, and variations in range of 19.42 to 78.42%. Women aged 25–34 years old (AOR = 1.26 95%CI: 1.21, 1.32) and 35–49 years (AOR = 1.33 95%CI: 1.27, 1.40), attending primary education (AOR = 1.26, 95% CI: 1.20, 1.32), secondary education (AOR = 1.50, 95% CI: 1.43, 1.58) and higher education (AOR = 2.01, 95% CI: 1.84, 2.19), having media exposure (AOR = 1.12, 95%CI: 1.07, 1.16), utilizing IUD (AOR = 1.98, 95%CI: 1.79, 2.19), injectable (AOR = 1.29, 95%CI: 1.23, 1.36) and implants (AOR = 1.70, 95%CI: 1.61, 1.79), survey year 2016–2020 (AOR = 1.38, 95%CI: 1.31, 1.44), women from lower middle (AOR = 1.25, 95%CI: 1.19, 1.31) and upper middle income level countries (AOR = 1.37, 95%CI: 1.23, 1.53) were associated with increased odds of informed choice of contraceptive methods. While, women who accessed contraceptives from private clinics (AOR = 0.64, 95%CI: 0.61, 0.67), pharmacies (AOR = 0.37, 95%CI: 0.35, 0.40), and others (AOR = 0.47, 95%CI: 0.43, 0.52), women in East Africa (AOR = 0.70, 95% CI: 0.67, 0.73), Central Africa (AOR = 0.52, 95% CI: 0.47, 0.57), and South Africa (AOR = 0.36, 95% CI: 0.32, 0.40) were associated with decreased odds of informed choice of contraceptive methods.

**Conclusion:**

The pooled prevalence of informed choice of contraceptive methods in Sub-Saharan Africa is low with high disparities among the countries. Enhancing maternal education and media exposure, providing greater concern for the source of contraceptive methods, and strengthening the economic status of the country are recommended to enhance informed choice of contraceptive methods.

## Introduction

The 1994 International Conference on Population and Development in Cairo declared that access to family planning should be a core part of reproductive and sexual health care services (1). Family planning encompasses the services, policies, information, attitudes, practices, and commodities, including contraceptives, that give users the ability to avoid unintended pregnancy and choose whether and/or when to have a child (2). Basically, access to contraceptive information and services is a human right, and the quality of contraceptive information and services reinforces people's freedom to determine the number and spacing of their children and offers a range of potential benefits, including maternal and child health (3). Informed choice is defined as the right of individuals or couples to make a voluntary, well-considered decision or choice that is based on options, information, and understanding (4, 5).

Many contraceptive users discontinue their contraceptive methods or fail to use them appropriately (6, 7) and unintended pregnancy is a public health problem in Sub-Saharan Africa (SSA) (8, 9). Informed choice of contraceptive methods is an indicator of the quality of family planning services (10). Evidence shows that informed choice of contraceptive methods decreases the risk of contraceptive method discontinuation rate, unmet need for contraceptives, unintended pregnancies, and induced abortions (11–14). Because it has the potential to play a key role in supporting individuals to select a method that matches their needs and expectations to promote effective contraceptive use (15). Furthermore, family planning providers' quality of counseling on all of the contraceptive methods, including addressing misconceptions and fears that exist about modern contraceptives and emphasizing the benefits of contraceptive methods during counseling, increased the contraceptive continuation rate (16, 17).

Evidence on the prevalence of informed choice of contraceptive methods across different parts of the world showed varying prevalence. A study done in India showed that 15.6% of contraceptive users had informed choice (18). A study conducted in Bangladesh found that 22% of women had an informed choice of contraceptive methods (19). Findings from Kenya showed that more than half (55.5%) of women had an informed choice of contraceptive methods (20). In Ethiopia, a study conducted based on the 2016 Ethiopian Demographic and Health Survey (EDHS) reported the prevalence of informed choice of contraceptive methods was 36.2% (21).

Previous studies on informed choice of contraceptive methods revealed that maternal age, place of residence, type of contraceptive use, wealth status, media exposure, maternal education, husband's occupation, source of contraceptive method, and visited health facility in the last 12 months were significantly associated with informed choice of contraceptive methods (12, 18, 21, 22).

Globally, different strategies have been applied to increase contraceptive utilization, but the prevalence of contraceptive methods in SSA remains unacceptably low (23) and the misconception of contraceptive side effects is a major public health problem (24). Even though studies have been conducted on the utilization, unmet need, and discontinuation of contraceptive methods in SSA countries, to our knowledge, evidence on informed choice of contraceptive methods in SSA countries is scarce. Therefore, this study aimed to investigate the pooled prevalence and determinants of informed choice of contraceptive methods among women aged 15–49 who are currently using selected modern contraceptive methods (pills, Intrauterine Device (IUD), injectable, female sterilization, and implants) in SSA. The study findings could help to inform the design of evidence-based public health interventions for enhancing informed choice of contraceptive methods and subsequently reduce the method discontinuation rate in SSA.

## Methods

### Data source

The data used in this study were the most recent Demographic and Health Surveys (DHS) data compiled in the 32 SSA countries (Angola, Burkina-Faso, Benin, Burundi, Democratic Republic of Congo, Congo, Ivory Coast, Cameroon, Ethiopia, Gabon, Ghana, Gambia, Guinea, Kenya, Comoros, Liberia, Lesotho, Mali, Malawi, Nigeria, Niger, Namibia, Rwanda, Sierra Leone, Senegal, Chad, Togo, Tanzania, Uganda, South Africa, Zambia, and Zimbabwe) from 2010 to 2019/2020. The data were derived from the measure DHS program. These datasets were merged together to determine the pooled prevalence and determinants of informed choice of contraceptive methods across the SSA countries. The DHS data is a nationwide representative survey and it has different datasets (men, women, birth, children, and household datasets). For this study, we used the individual data set (IR file). The DHS used two stages of stratified sampling technique to select the study participants. We pooled the DHS surveys conducted in the 32 SSA countries and a total weighted sample of 65,487 women aged 15–49 who are currently using selected modern contraceptive methods (pill, IUD, injectable, female sterilization, and implants) was included in the study.

### Study variables

#### Outcome variable

The outcome variable for this study was informed choice of contraceptive methods, which was a binary outcome variable coded as “0” if a woman did not receive informed choice of contraceptive methods, and “1” if she did receive informed choice of contraceptive methods. The variable was generated using the three questions. The three questions were: (1) “Were you told about possible side effects or problems you might have with the method?” (2) “Were you told what to do if you experience any side effects?” and (3) “Were you told about other methods of family planning?” Answers were coded as 1 = Yes and 0 = No, then categorized as a woman received informed choice of contraceptive methods if they answered all three questions, and otherwise they did not receive an informed choice of contraceptive methods (18, 25).

#### Explanatory variables

The independent variables were classified as individual level variables such as age, marital status, and maternal educational status, husband educational status, maternal occupation, husband occupation, media exposure status, household wealth index, internet use, visiting health facilities in the last 12 months, source of contraceptive method, and type of contraceptive use. Whereas, community level variables, such as residence, region in SSA countries, DHS survey year, and country income level.

Media exposure was calculated from three variables; listening to the radio, reading newspapers, and watching television. If a woman were exposed to at least one type of media, she was considered as exposed to media (26).

The country income level was categorized as low income, lower middle income, and upper-middle-income country based on the World Bank List of Economies since 2019 (27).

### Data management and analysis

After we accessed the data from the DHS program, we cleaned the data, carried out cross-tabulation, and calculated descriptive and summary statistics using STATA version 14 software. Before inferential analysis, we applied weighting using sampling weight, primary sampling unit, and strata to restore the representativeness of the survey and to get reliable statistical estimates. The pooled prevalence of informed choice of contraceptive method across countries from 2010 to 2019/2020 was done using a metan STATA command and presented in a forest plot with 95% Confidence Interval (CI). Besides, further subgroup analyses were done to minimize the heterogeneity between studies using regions in SSA countries and the DHS survey year. The multilevel binary logistic regression analysis were used to determine the association between the likelihood of informed choice of contraceptive methods and explanatory variables at both individual and community levels. After bivariable analysis, variables with a *p*-value < 0.2 were included in the multivariable multilevel logistic regression model. Adjusted Odds Ratios (AOR) with a 95% CI and a *p*-value < 0.05 in the multivariable multilevel logistic model were used to declare significant determinants of informed choice of contraceptive methods.

### Model building

The DHS data has a hierarchical nature. Hence, women are nested within a cluster, and we anticipate that women within the same cluster may be more similar to each other than women in the rest of the country. This implies that advanced models need to take into account the between cluster variability. Therefore, we used the mixed-effect logistic regression analysis method. Moreover, model comparison and fitness were assessed based on the Intra-class Correlation Coefficient (ICC), Median Odds Ratio (MOR), Proportional Change in Variance (PCV), and deviance (-2LLR) values. The calculation for MOR and PCV is as follows;

${{\;MOR=e}^{0.95}}^{\sqrt{VA}}$ (28) where; VA is the area level variance
$PCV={\frac{Vnull-VA}{V\;null}}^{*}100\%\;$ (28) where; Vnull = variance of the initial model, and VA = variance of the final model.

Generally, in this study, four models were fitted: the first model was a null model (model without the independent variables), which was used to check the variability of informed choice of contraceptive methods in the cluster. The second (model I) and third (model II) models, which contain individual-level variables and community-level variables, respectively. The final model (model III), which contains both individual and community-level variables simultaneously. We used deviance to select the best fit model for the data. The final model (model III) was selected because of its lowest deviance value (**Table 3**).

### Ethics consideration

Ethical approval and participant consent were not necessary for this study since it was a secondary data analysis based on the publically available DHS data. We requested to access the data from the measure DHS program and permission was obtained to download and use the data for this study. There are no personal identifier in the data files.

## Result

### Descriptive characteristics of the study participants

A total of 65,487 reproductive age women who were currently using selected modern contraceptive methods were included in this study (Table 1). Of these, 39,849 (60.85%) were from rural areas, and 37,103 (56.66%) from East Africa, 19,356 (29.56%) from West Africa, 3,428 (5.23%) in Central Africa, and 5,600 (8.55%) in Southern Africa. Regarding the age of women, 29,727 (45.39%) of women were under the age group of 25–34 years. The majority (80.44%) and (71.34%) of women were married and employed, respectively. Nearly half (46.94%) of the women were from rich household wealth status, and the majority (74.30%) of women had media exposure. About 50,570 (77.22%) and 31,605 (48.26%) of women had access to family planning from a government facilities and utilized an injectable type of contraceptive method, respectively (Table 2).

**Table 1:** Sample size calculation (country and DHS-year wise) in the study of pooled prevalence and determinants of informed choice of contraceptive methods among reproductive age women in Sub Saharan Africa (2010–2020 DHS).

**Sub Saharan region**	**Countries**	**Sample size (*n* = 65,487)**	**DHS year**
West Africa	Benin Burkina Faso Ivory Coast Gambia Ghana Guinea Liberia Mali Niger Nigeria Senegal Sierra Leone Togo	1,740 1,335 732 1,316 1,284 689 1,741 1,490 2,526 807 3,353 1,343 1000	2017/18 2011 2011/12 2019/20 2014 2018 2019/20 2018 2012 2018 2019 2019 2013/14
East Africa	Burundi Comoros Ethiopia Kenya Malawi Rwanda Tanzania Uganda Zambia Zimbabwe	2,068 3,453 4,322 352 8,695 4,170 2,669 3,841 3,797 3,736	2016/17 2012 2016 2014 2015/16 2019/2020 2015/16 2016 2018 2015
Central Africa	Angola Cameroon Chad DR Congo Congo Gabon	827 447 470 831 336 517	2015/16 2018 2014/15 2013/14 2011/12 2012
Southern Africa	Lesotho Namibia South Africa	1,563 2,174 1,863	2014 2013 2016
Total sample size	65,487		

**Table 2:** Descriptive characteristics of the study participants in Sub-Saharan Africa.

**Variable**	**Categories**	**Informed choice of contraceptive method**		**Total weighted frequency (%)**
		**Yes (%)**	**No (%)**	
Age	15–24 25–34 ≥35	8,920 (50.20) 16,461 (55.37) 10,027 (55.74)	8,849 (51.07) 13,266 (45.54) 7,964 (45.38)	17,769 (27.13) 29,727 (45.39) 17,991 (27.47)
Marital status	Unmarried Married Widowed/divorced/separated	3,969 (51.48) 28,634 (54.36) 2,805 (54.99)	3,741 (49.52) 24,041 (45.64) 2,297 (45.01)	7,710 (11.77) 52,675 (80.44) 5,102 (7.97)
Maternal educational status	No education Primary Secondary Higher	6,960 (51.78) 13,965 (53.42) 12,224 (54.93) 2,261 (61.92)	6,480 (48.22) 12,176 (46.58) 10,031 (45.07) 1,390 (38.08)	13,440 (20.52) 26,141 (39.92) 22,255 (33.98) 3,651 (5.57)
Husband education status	No education Primary Secondary Higher	5,851 (51.13) 10,218 (52.82) 10,039 (55.82) 3,025 (60.22)	5,592 (48.87) 9,127 (47.18) 7,946 (44.18) 1,998 (39.78)	11,443 (21.27) 19,345 (35.96) 17,985 (33.43) 5,023 (9.34)
Maternal occupation status	Not working Working	9,399 (50.08) 26,010 (55.67)	9,371 (49.92) 20,707 (44.33)	18,770 (28.66) 46,717 (71.34)
Husband occupation status	Not working Working	1,680 (52.50) 27,410 (54.53)	1,520 (47.50) 22,859 (45.47)	3,200 (5.99) 50,269 (94.01)
Media exposure	No Yes	8,807 (52.33) 26,602 (54.67)	8,021 (47.67) 22,057 (45.33)	16,828 (25.70) 48,659 (74.30)
Wealth status	Poor Middle Rich	11,626 (53.49) 6,901(53.02) 16,882 (54.92)	10,109 (46.51) 6,113 (46.98) 13,856 (45.08)	21,735 (33.19) 13,014 (19.87) 30,738 (46.94)
Internet use	No Yes	31,132 (53.25) 4,277 (60.91)	27,333 (46.75) 2,745 (39.09)	58,465 (89.28) 7,022 (10.72)
Visiting health facility in the last 12 months	No Yes	9,790 (49.27) 25,618 (56.16)	10,080 (50.73) 19,999 (43.84)	19,870 (30.34) 45,617 (69.66)
Source of contraceptive method	Government NGO Private Pharmacy Others	29,031 (57.41) 753 (55.87) 3,520 (48.23) 1,435 (32.61) 670 (35.83)	21,539 (42.59) 594 (44.13) 3,778 (51.77) 2,966 (67.39) 1,201 (64.17)	50,570 (77.22) 1,347 (2.06) 7,298 (11.14) 4,401 (6.72) 1,871 (2.86)
Type of contraceptive method	Pills IUD Injectable Sterilization Implant	5,196 (42.96) 1,544 (67.82) 16,647 (52.67) 1,032 (45.86) 10,989 (63.67)	6,899 (57.04) 733 (32.18) 14,958 (47.33) 1,219 (54.14) 6,270 (36.33)	12,095 (18.47) 2,277 (3.48) 31,605 (48.26) 2,251 (3.44) 17,259 (26.35)
**Community level variables** Residence	Urban Rural	14,018 (54.68) 21,390 (53.68)	11,620 (45.32) 18,459 (46.32)	25,638 (39.15) 39,849 (60.85)
Region of Sub Saharan Africa countries	West Africa East Africa Central Africa Southern Africa	11,485 (59.34) 20,082 (54.12) 1,537 (44.83) 2,305 (41.16)	7,871 (40.66) 17,021 (45.88) 1,891 (55.17) 3,295 (58.84)	19,356 (29.56) 37,103 (56.66) 3,428 (5.23) 5,600 (8.55)
Survey year	2010–2015 2016–2020	7,370 (46.72) 28,039 (56.40)	8,404 (53.28) 21,674 (43.60)	15,774 (24.09) 49,713 (75.91)
Country income level	Low income Lower middle Upper middle	24,549 (54.88) 8,542 (54.91) 2,318 (44.58)	20,181 (45.12) 7,015 (45.09) 2,882 (55.42)	44,730 (68.93) 15,557 (23.76) 5,200 (7.94)

### Pooled prevalence of informed choice of contraceptive methods in SSA

**Figure 1:**
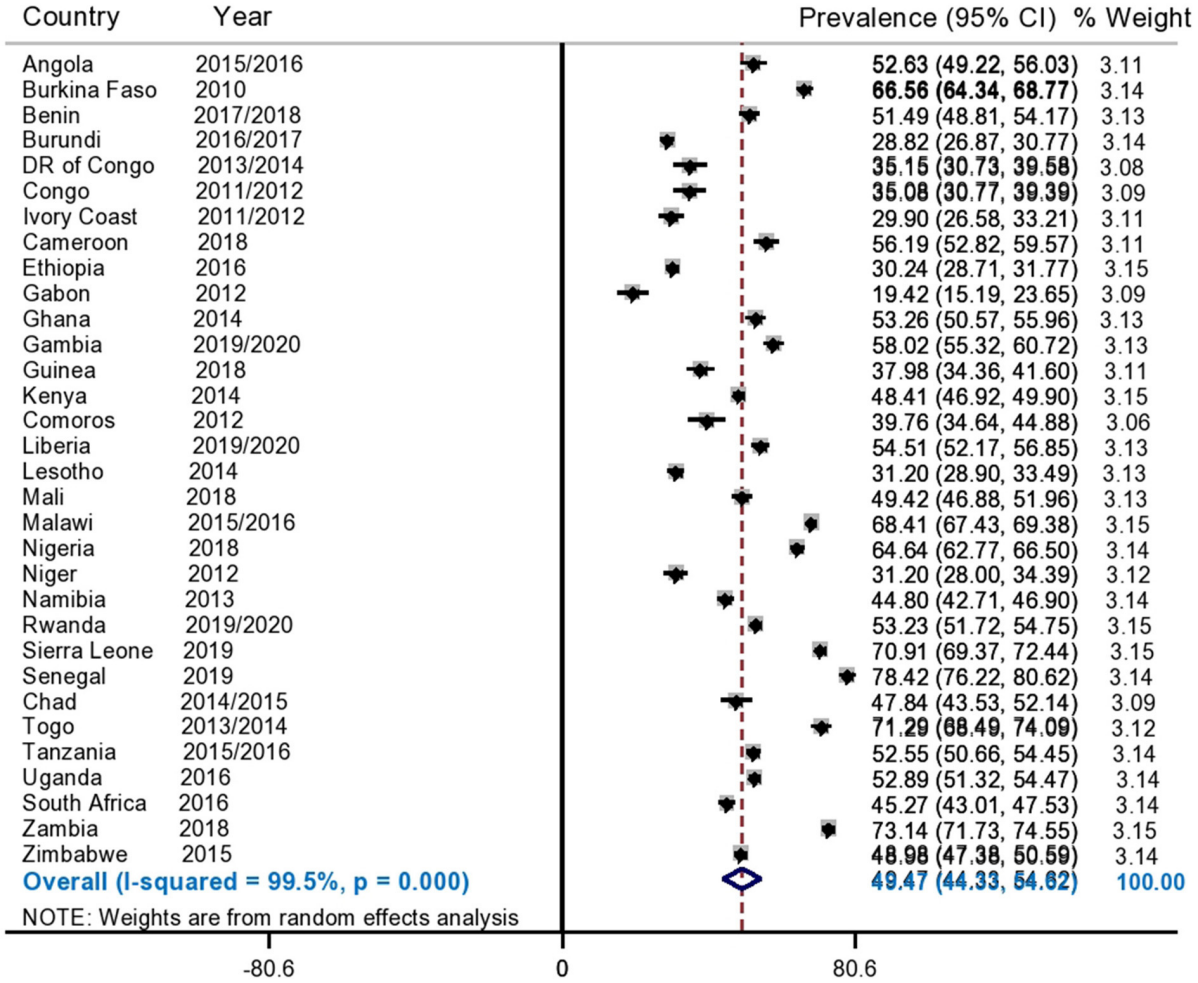
Forest plot showed that, the pooled prevalence of informed choice of contraceptive methods among reproductive age women in SSA.

The pooled prevalence of informed choice of contraceptive methods in SSA countries was found to be 49.47% (95%CI: 44.33, 54.62%) with *I*^2^ = 99.5% and variations among countries were also observed ranges from 19.42% (95%CI: 15.19, 23.65%) in Gabon to 78.42% (95%CI: 76.22, 80.62%) in Senegal (Figure 1).

**Figure 2:**
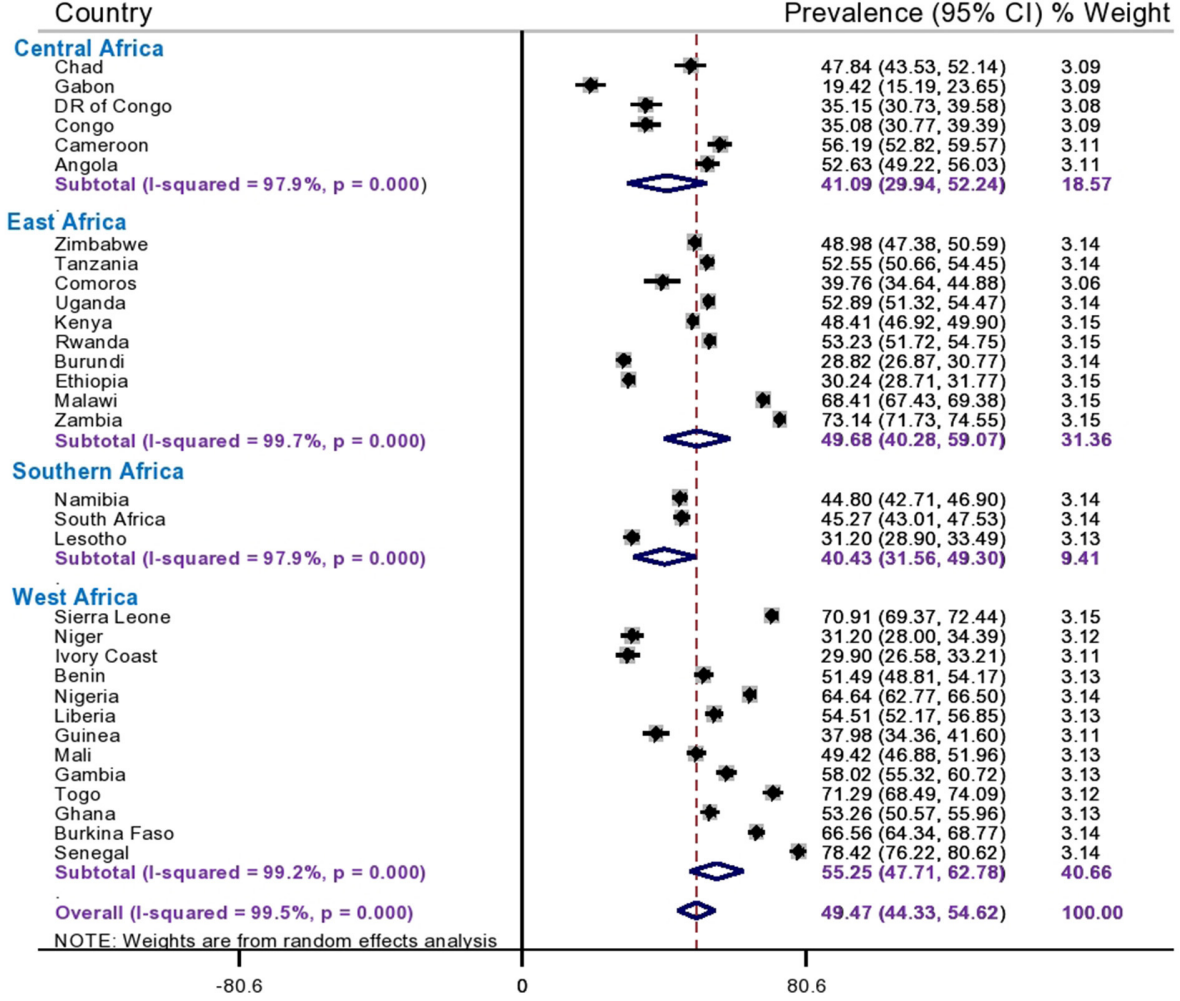
Subgroup analysis of informed choice of contraceptive methods by region in Sub-Saharan Africa.

**Figure 3:**
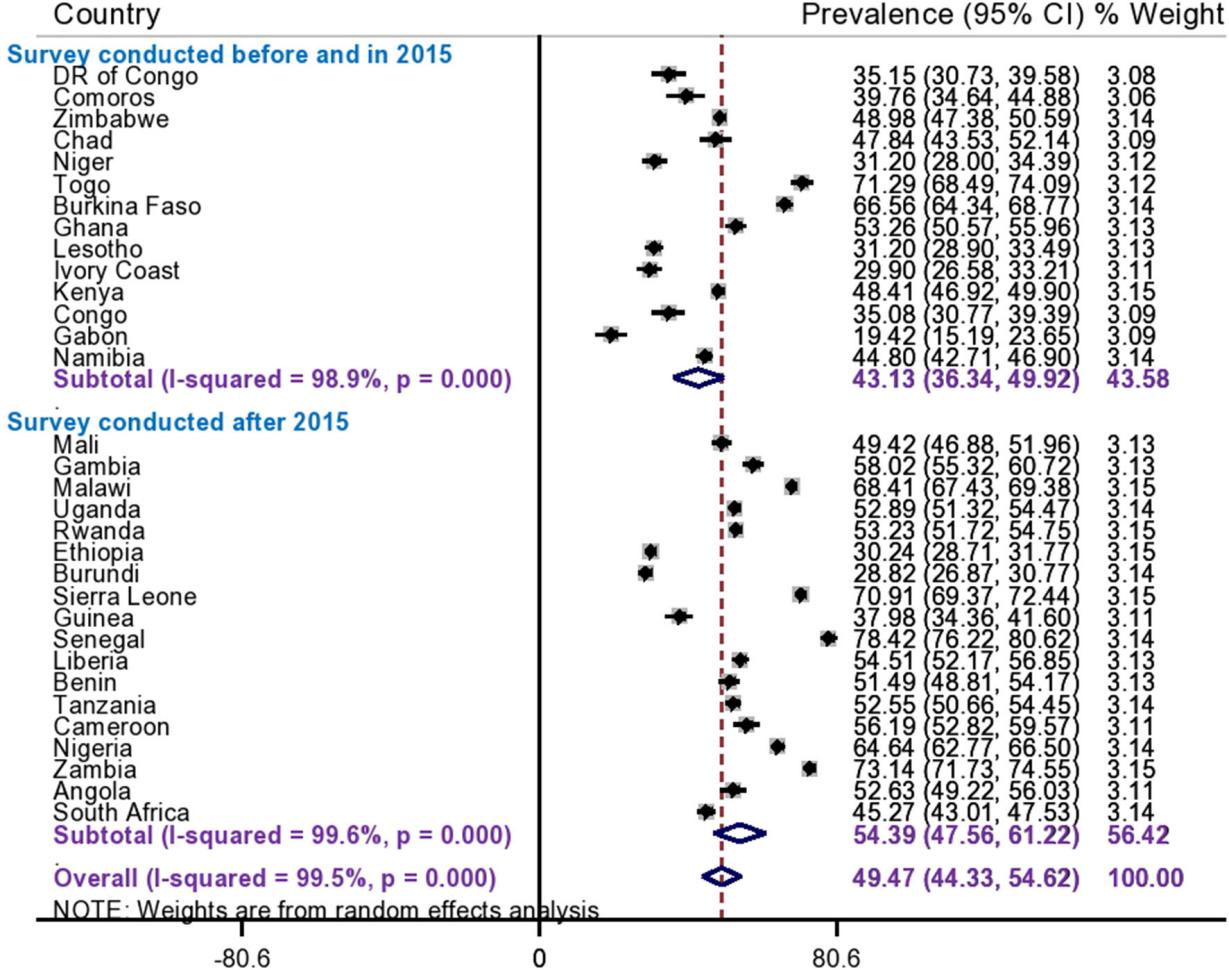
Subgroup analysis of informed choice of contraceptive methods by survey year in Sub Saharan Africa.

Since the *I*^2^ value was large, which shows that the true variability (the variability is not by chance) of informed choice of contraceptive methods between countries, then to treat this heterogeneity effect further subgroup analyses were done based on the region of SSA and DHS survey year. Based on this, the subgroup analysis using regions of SSA countries shows that the pooled prevalence of informed choice of contraceptive methods ranges from 40.43% (95%CI: 31.56, 49.30%) in Southern African countries to 55.25% (95%CI: 47.71, 62.78) in West African countries (Figure 2). Besides, the pooled prevalence of informed choice of contraceptive methods across the DHS survey year was estimated. The pooled prevalence of informed choice of contraceptive methods was 43.13% (95%CI: 36.34, 49.92%) among countries whose DHS survey was conducted before and in 2015, whereas it was 54.39% (95%CI: 47.56, 61.22%) in countries whose DHS survey was conducted after 2015 (Figure 3).

### Individual and community level determinants of informed choice of contraceptive methods

#### Random effect analysis and model fitness

The random effect analysis revealed statistically significant variation in informed choice of contraceptive methods between clusters. The ICC value in the null model was 28%, which showed that about 28% of the total variability of informed choice of contraceptive methods among reproductive age (15–49) women was attributed to the between-cluster variability, whereas the remaining 72% of the total variability was explained by the between-individual difference. The PCV value in the final model (model III) was 20% and showed that the variation in informed choice of contraceptive methods was explained by the final model (both the individual and community level determinants). Moreover, the final model (model III) was the best-fitted model since it had the lowest deviance value (86,080) (Table 3).

**Table 3:** Random effect analysis result and model fitness.

**Parameter**	**Null model**	**Model I**	**Model II**	**Model III**
VA	1.26	1.20	1.05	1.01
ICC	0.28	0.27	0.24	0.23
MOR	2.90	2.83	2.65	2.60
PCV	Reff	0.05	0.17	0.20
Loglikelyhood ratio	−44892	−43472	−44317	−43040
Deviance	89,784	86,472	88,634	86,080

#### The mixed effect analysis result

In the final model of multilevel logistics regression analysis variables such as; maternal age, maternal education, media exposure, source of family planning, type of current contraceptive use, region of SSA countries, DHS year, and country income level were found to be determinants significantly associated with informed choice of contraceptive methods. Thus, the odds of having an informed choice of contraceptive methods among women aged 25–34 and 35–49 years old was 1.26 times (AOR = 1.26, 95%CI: 1.21, 1.32), and 1.33 times (AOR = 1.33, 95%CI: 1.27, 1.40) higher as compared to 15–24 years old women, respectively. Similarly, women who attended primary education, secondary, and higher education levels had 1.26 times (AOR = 1.26, 95% CI: 1.20, 1.32), 1.50 times (AOR = 1.50, 95% CI: 1.43, 1.58), and 2.01 times (AOR = 2.01, 95% CI: 1.84, 2.19) higher odds of informed choice of contraceptive methods than those who had no formal education, respectively. Women who had media exposure had 1.12 times (AOR = 1.12, 95%CI: 1.07, 1.16) higher odds of informed choice of contraceptive methods as compared to their counterparts.

Regarding the source of contraceptive methods, women who accessed family planning from private clinics, pharmacies, and others were 36% (AOR = 0.64, 95%CI: 0.61, 0.67), 63% (AOR = 0.37, 95%CI: 0.35, 0.40), and 53% (AOR = 0.47, 95%CI: 0.43, 0.52) less likely to have an informed choice of contraceptive methods as compared to those who accessed from government clinics, respectively. Whilst, women who utilized IUD, injectable, and implants had 1.98 (AOR = 1.98, 95%CI: 1.79, 2.19), 1.29 (AOR = 1.29, 95%CI: 1.23, 1.36), and 1.70 (AOR = 1.70, 95%CI: 1.61, 1.79) times higher odds of having an informed choice of contraceptive methods than women who utilized pill types of contraceptive methods, respectively.

Among community level variables, the odds of having an informed choice of contraceptive methods among women in East Africa, Central Africa and South Africa were decreased by 30% (AOR = 0.70, 95% CI: 0.67, 0.73), 48% (AOR = 0.52, 95% CI: 0.47, 0.57), and 64% (AOR = 0.36, 95% CI: 0.32, 0.40) compared to women in West Africa, respectively. Moreover, women from lower middle and upper middle income level countries had 1.25 (AOR = 1.25, 95%CI: 1.19, 1.31) and 1.37 (AOR = 1.37, 95%CI: 1.23, 1.53) times higher odds of informed choice of contraceptive methods as compared to women from low-income countries, respectively (Table 4).

**Table 4:** Individual and community-level determinants of informed choice of contraceptive methods in sub-Saharan Africa.

**Variable**	**Null model**	**Model I AOR (95%CI)**	**Model II AOR (95%CI)**	**Model III AOR (95%CI)**
**Individual level variables**				
**Age**				
15–24		1		1
25–34		1.21 (1.16, 1.26)		1.26 (1.21, 1.32)***
35–49		1.29 (1.23, 1.35)		1.33 (1.27, 1.40)***
**Marital status**				
Unmarried		1		1
Married		1.04 (0.99, 1.10)		1.01 (0.95, 1.06)
Widowed/divorced/separated		1.16 (0.98, 1.14)		1.06 (0.98, 1.15)
**Maternal education status**				
No education		1		1
Primary		1.11 (1.06, 1.56)		1.26 (1.20, 1.32)***
Secondary		1.31 (1.25, 1.37)		1.50 (1.43, 1.58)***
Higher		1.74 (1.60, 1.89)		2.01 (1.84, 2.19)***
**Maternal occupation status**				
Not employed		1		1
Employed		1.14 (1.09, 1.18)		1.03 (0.99, 1.07)
**Wealth status**				
Poor		1		1
Middle		0.97 (0.93, 1.01)		0.96 (0.88, 1.02)
Rich		1.06 (1.01, 1.10)		0.98 (0.93, 1.03)
**Media exposure**				
No		1		1
Yes		1.08 (1.04, 1.13)		1.12 (1.07, 1.16)***
**Source of contraceptive method**				
Government clinic		1		1
NGO		0.92 (0.82, 1.04)		1.01 (0.90, 1.14)
Private clinic		0.64(0.61, 0.68)		0.64 (0.61,
Pharmacy		0.42 (0.39, 0.45)		0.67)***
Others		0.48 (0.43, 0.53)		0.37 (0.35, 0.40)***
				0.47 (0.43, 0.52)***
**Type of contraceptive use**				
Pills		1		1
IUD		2.14 (1.94, 2.37)		1.98 (1.79,
Injectable		1.30 (1.23, 1.36)		2.19)***
Sterilization		0.94 (0.86, 1.02)		1.29 (1.23, 1.36)**
Implant		1.88 (1.78, 1.98)		0.97 (0.89, 1.06) 1.70 (1.61, 1.79)***
**Community level variables**				
**Residence**				
Urban			1	1
Rural			0.96 (0.92, 0.99)	0.95 (0.89, 1.01)
**Sub Saharan Africa region**				
West Africa			1	1
East Africa			0.75 (0.72, 0.77)	0.70 (0.67, 0.73)***
Central Africa			0.51 (0.47, 0.56)	0.52 (0.47, 0.57)***
Southern Africa			0.42 (0.38, 0.46)	0.36 (0.32, 0.40)***
**Survey year**				
2010–2015			1	1
2016–2020			1.52 (1.46, 1.59)	1.38 (1.31, 1.44)***
**Country income level**				
Low income			1	1
Lower middle income			1.31 (1.25, 1.37)	1.25 (1.19, 1.31)**
Upper middle income			1.38 (1.24, 1.53)	1.37 (1.23, 1.53)***

**P-value < 0.01, ***P-value < 0.001.

## Discussion

This study aimed to assess the pooled prevalence of informed choice of contraceptive methods and its determinants among women aged 15–49 who are currently using selected modern contraceptive methods (pill, IUD, injectable, female sterilization, and implants) in SSA countries. In this study, the pooled prevalence of informed choice of contraceptive methods in 32 SSA countries was 49.47% (95%CI: 44.33, 54.62%). This finding is lower than a study done in Kenya (20). The possible reason for the observed variation might be the difference in study setting. In Kenya, the study was conducted in urban setting, which might lead to an increase in the prevalence of informed choice of contraceptive methods because women who reside in urban areas can access more information about contraceptive methods in different ways. In contrast, our study was conducted among women who reside in urban and rural areas. However, the finding of our study is higher than studies done in Ethiopia (21) and India (18). Possible justification for this discrepancy could be the difference in the study period and the study population.

In the multivariable logistic regression, we found that maternal age, maternal education, media exposure, source of contraceptive method, type of contraceptive use, SSA region, DHS survey year, and country income level were significantly associated with the higher odds of informed choice of contraceptive methods. Whereas, source of family planning and SSA region were significantly associated with lower odds of informed choice of contraceptive methods. In this study, as women's age increases, the chance of having an informed choice of contraceptive methods also increases. This result was consistent with a study done in India (18). This could be due to healthcare providers' discriminatory attitudes toward younger women (29).

The study at hand also revealed that women who attained primary, secondary, and higher education had higher odds of having an informed choice of contraceptive methods compared to women who did not have formal education, respectively. This is in line with studies conducted elsewhere (18, 21, 25, 30). The possible justification might be that educated women may be more likely to engage in providers' conversations and understand the counseling information they receive from their providers (22, 31). Besides, they were better able to retain the information or they obtained it from other sources like media. This finding shows that the importance of women's education for enhancing informed choice of contraceptive methods in SSA.

In our study, media exposure is one of the most important determinants of informed choice of contraceptive methods. Women who had media exposure were more likely to have an informed choice of contraceptive methods than women who had no media exposure. The possible reasons for this finding could be that women who had media exposure easily retained information about different types of contraceptive methods and their side effects, which enabled them to make an informed choice of contraceptive methods (32).

Women receiving contraceptive methods from private clinics, pharmacies, and others had significantly lower informed choice of contraceptive methods than those who received contraceptives from government clinics. This result was supported by a study conducted in Ethiopia (30). This might be due to the fact that both private clinics and pharmacies may have no family planning guidelines/protocols and trained family planning providers for counseling about the three components of informed choice as compared with government clinics (33).

Women who utilized IUD, injectable, and implants had higher odds of having an informed choice of contraceptive methods than women who utilized pill-type contraceptive methods, respectively. The possible explanation could be that healthcare workers might be given more attention to provide good information to those long-acting contraceptive users to promote its coverage because healthcare workers have a major role in contraceptive method uptake (23, 34).

Furthermore, country income level and DHS survey year were significant determinants of informed choice of contraceptive methods. Women from lower middle and upper middle income level countries, as well as those from survey year after 2015, were more likely to have an informed choice of contraceptive methods than women from lower-income level countries and survey year ≤ 2015. This might be due to the fact that women living in better-income countries have good access to basic quality maternal health care services compared to their counterparts (35). Besides, toward the later survey years, family planning services might be increased because it's a government concern to achieve the sustainable development goal target 3.7.

This study has both strengths and limitations. This study was based on nationally representative data with weighted and multilevel statistical analysis model, which revealed a reliable estimate and standard error. In addition, this study was based on a large sample size that had adequate power to detect the true effect of the explanatory variables. Due to this, the findings of this study can help policymakers and public and private organizations take appropriate interventions to enhance informed choice of contraceptive methods. However, our study was not without limitations. The heterogeneity of the pooled estimate of informed choice of contraceptive methods was not managed using further subgroup analysis. Since it was based on cross-sectional data, we are unable to establish a causal relationship between informed choice of contraceptive methods and the identified independent variables. Since we use the secondary data recall biases and social desirability biases might be expected. Furthermore, we used DHS conducted during the previous 10 years, and there could be changes in informed choice of contraceptive methods over time.

## Conclusions

In conclusion, the pooled prevalence of informed choice of contraceptive methods among reproductive-aged (15–49) women who were currently using selected modern contraceptive methods (pill, IUD, injectable, female sterilization, and implants) in sub-Saharan Africa is low with a substantial heterogeneity. Maternal age, maternal education, media exposure, source of contraceptive method, type of contraceptive use, SSA region, DHS survey year, and country income level were found to be significant determinants of informed choice of contraceptive methods. Health care providers shall give special attention for those group of women with the identified determinants to increase their informed choice of contraceptive methods. Enhancing maternal education and media exposure, providing greater concern for the source of contraceptive methods (private clinics, pharmacies and other) to deliver essential information for users to make an informed choice, and strengthening the economic status of the country are recommended to enhance informed choice of contraceptive methods.

## Data availability statement

The original contributions presented in the study are included in the article/supplementary material, further inquiries can be directed to the corresponding author/s.

## Ethics statement

We requested to access the data from the measure DHS program and permission was obtained to download and use the data for this study. Since the study is a secondary data analysis based on DHS data, consent to participate is not applicable.

## Author contributions

The conception of the work, design of the work, acquisition of data, analysis, and interpretation of data was done by NT, DB, TH, and MA. Data curation, drafting the article, revising it critically for intellectual content, validation and final approval of the version to be published was done by NT, TH, MA, DB, ME, FA, ST, and MG. All authors read and approved the final manuscript.

## Conflict of interest

The authors declare that the research was conducted in the absence of any commercial or financial relationships that could be construed as a potential conflict of interest.

## Publisher's note

All claims expressed in this article are solely those of the authors and do not necessarily represent those of their affiliated organizations, or those of the publisher, the editors and the reviewers. Any product that may be evaluated in this article, or claim that may be made by its manufacturer, is not guaranteed or endorsed by the publisher.

## Abbreviations

AOR: Adjusted Odds Ratio
CI: Confidence Interval
DHS: Demographic and Health Survey
EDHS: Ethiopia Demographic and Health Survey
ICC: Intra-class Correlation Coefficient
IUD: Intrauterine Device
MOR: Median Odds Ratio
PCV: Proportional Change in Variance
SSA: Sub-Saharan Africa.
